# “Machine replacement” or “job creation”: How does artificial intelligence impact employment patterns in China's manufacturing industry?

**DOI:** 10.3389/frai.2024.1337264

**Published:** 2024-03-06

**Authors:** Qingqing Huo, Jing Ruan, Yan Cui

**Affiliations:** ^1^School of Statistics, Capital University of Economics and Business, Beijing, China; ^2^School of Data Science, Capital University of Economics and Business, Beijing, China; ^3^Chinese Academy of Labour and Social Security, Beijing, China

**Keywords:** AI, total employment, employment structure, employment quality, industry spillover

## Abstract

Artificial intelligence (AI), as an important engine for promoting high-quality economic development, should not be overlooked in terms of its impact on the employment of the labor force while promoting the digital and intelligent transformation of industries. In the face of the complex international environment and non-systemic shocks, it is of great significance to explore whether it is “machine replacement” or “job creation” in the process of the integration of AI and industry, as well as the impact of technological progress on the employment pattern of the labor force, in order to promote the economic development, respond to and solve the employment problem. It is of great significance to promote economic development and cope with and solve the employment problem. Based on the task model, this paper analyses the mechanism of the impact of AI on the employment pattern of manufacturing industry. Meanwhile, based on the provincial panel data of China's manufacturing industry from 2011 to 2020, it empirically examines the impact of AI on the total employment, employment structure and employment quality of the labor force, and analyses the multiple responses of AI on the employment pattern of the manufacturing industry. The study shows that: Firstly, the level of development of AI and the total amount of employment is a positive U-shaped relationship, the short term is dominated by the substitution effect, and the long term is dominated by the creation effect; Secondly, with regard to the employment structure, low-skilled labor is more likely to be replaced. The financial, accommodation and catering industries are relatively less affected by the spillover effects of the manufacturing industry; Third, with regard to the employment quality, the gap between urban and rural incomes has eased, with per capita net income of rural residents rising to a higher degree than per capita disposable income of urban residents. Thus, in order to further address the impact of AI on the employment patterns of the labor force, the level of AI development should be increased while expanding employment channels, paying attention to labor force skills training, reinforcing the leading role of developed regions, and accelerating regional integration and urban-rural integration, so as to share the dividends of technological progress.

## 1 Introduction

“The new round of scientific and technological revolutions and industrial changes have given a strong boost to economic development, and have also had a profound impact on employment and income distribution, including a number of negative impacts, which need to be effectively dealt with and resolved” (Xi, 2021). Along with the high-quality development of the economy, AI technologies such as machine learning and natural language processing have infiltrated all walks of life from the Internet. However, the manufacturing industry, as the lifeblood of China's economy and the foundation of the country, plays an important role in accelerating the upgrading of the industrial chain and the supply chain, solidifying the main position of the domestic macrocycle, and enhancing the ability of the international macrocycle to drive the country. China has also put forward a number of strategic plans for developing intelligent manufacturing. For example, in May 2015, the State Council issued Made in China 2025 to accelerate the deep integration of a new generation of information technology and manufacturing. In July 2017, the State Council issued the New Generation AI Development Plan, which focuses on the major needs of a strong manufacturing country and promotes full life-cycle manufacturing. In August 2019, the Ministry of Science and Technology, with cities as the main carrier, proposed the layout and construction of about 20 pilot areas by 2023, optimizing urban governance and leading the development of county economies. In November 2021, the Ministry of Industry and Information Technology and other departments proposed to build a number of factories with strong demonstration and high technical level by the end of the 14th Five-Year Plan, etc. Currently, the application of AI in manufacturing promotes high-quality economic development, reduces labor costs, improves product quality, and promotes the transformation of manufacturing to intelligence, which is a key step toward adapting to future economic needs and achieving sustainable development.

At the same time, in the face of a complex international environment and non-systemic shocks, the government and the public have become more concerned about employment and unemployment. The Future Jobs Report 2023, published by the World Economic Forum, mentions that “analytical and creative thinking, as well as AI and big data capabilities, will be the most in-demand skills over the next five years. Technological innovation and digital transformation are bringing opportunities to the market, with more than half of respondents expecting big data-related jobs to grow. But at the same time, some jobs will be threatened by AI, with nearly a quarter expected to potentially disappear.” So, in the process of convergence between AI and industry, will it be a case of “machine for man” or “job creation”? To what extent do technological advances in AI affect the labor force's employment patterns? Are there significant differences between heterogeneous groups? These questions require further research.

This study takes the manufacturing industry panel from 2011 to 2020 as the sample data and conducts theoretical analyses and empirical tests on AI affecting the employment pattern of the labor force based on the overall and regional perspectives from the three aspects of the labor force's total employment, employment structure, and employment quality. The innovation lies in the following: First, starting from the factors of labor and capital, combining with the task model, taking the production process of a single-sector economy as an example, building a logical framework of AI and labor employment pattern, improving the CES production function, and comprehensively and clearly understanding the substitution effect and the creation effect turnover from the micro perspective; Second, based on the previous study of the total labor employment volume and the structure of the employment volume, it is to explore the quality of the employment of the labor force from the perspective of the income. Based on the income level of urban and rural residents, it empirically analyses whether the development of AI is conducive to the improvement of the income level of residents and whether it can alleviate the urban–rural income gap and improve the quality of employment to promote the common prosperity of all the people; Third, unlike the previous standard of dividing the four major plates into regions, this study divides them into eight comprehensive economic zones for the analysis of heterogeneity, which is more targeted to promote regional integration and high-quality economic development. This study is divided into eight comprehensive economic zones for heterogeneous analysis, which is more targeted to promote regional integration and high-quality economic development.

## 2 Literature review

There are three main views on the impact of AI on the total employment of the labor force. The first view is that AI has a substitution effect on labor force employment. Frey and Osborne (2017) used data from the US Department of Labor to examine the role of AI and levels of automation on job substitution and found that nearly half of all jobs are at high risk of being replaced. Chiacchio et al. (2018) estimated that employment declines by 0.16–0.20 percentage points when a robot is added per 1,000 workers, with the substitution effect dominating. Yan et al. (2020) found that for every 1 percentage point rise in robots, labor force jobs fell by 4.6 percentage points. He et al. (2023) regarded the side-by-side collaboration between industrial robots and labor force as a new type of labor force form and believed that the influence of industrial robots on the labor force is mainly manifested as the substitution effect. The second view is that AI has a job-creating effect on labor employment. Berg et al. (2018) argued that industrial robots have led to a significant increase in labor productivity and labor demand, creating many new jobs. Dauth et al. (2021), in their analysis of the impact of robots on the German cross-industry and labor market, found no evidence of a shrinking employment scale due to robots. The overall decline in manufacturing employment and jobs was offset by additional jobs in the service sector, and the use of robots can significantly increase overall employment levels. The third view is that the impact of AI on labor employment depends on a combined comparison of substitution and creation effects. Acemoglu and Restrepo (2019) built a model of tasks. On the one hand, automation generates a substitution effect that shifts the allocation of tasks to factors of production relative to labor, and on the other hand, the introduction of new tasks generates a creation effect. Huang and Dong (2023) measured the coexistence of the substitution effect and creation effect of AI through numerical simulation, which can change the cross-sectoral flow of capital and labor factors, thus promoting the upgrading of industrial structure.

The impact of AI on the employment structure of the labor force cannot be ignored. On the one hand, for the different skills labor force, David (2017) argued that the middle-skilled labor force is more easily replaced, and its total labor force and wages are ranked the lowest compared to low-skilled and high-skilled, and there is a significant decrease. Sun and Hou (2019) similarly argued that the increase in the level of intelligence replaces middle- and high-school-educated skilled labor, and the demand for low- and high-educated labor increases, resulting in a bifurcated nature of employment patterns. However, Ramaswamy (2018) argued that the increase in automation and robotics applications does not lead to overall unemployment, but there is a decrease in the demand for low-skilled workers in routine jobs and an increase in the demand for new types of skilled workers or high-skilled workers. Autor and Salomons (2018) argued that technological advances may stimulate an increase in the market share of the industry for relatively capital-intensive firms, a decrease in the market share of industries with relatively labor-intensive firms, and a shift of low-skilled labor from capital-intensive to labor-intensive firms. Zhu and Li (2018) argued that the development of AI is accompanied by an increase in the demand for skilled labor, which leads to an overall increase in skill levels. On the other hand, for industry spillovers, Wang et al. (2017) argued that AI has a substitution effect on total employment while the risk of inter-industry crowding out also exists. Wang and Chang (2021), in their study of the impact of AI on the employment structure of the labor force, found that the development of AI is likely to lead to employment polarization and thus widen the income gap and accelerate the transfer of labor to the service industry.

In addition to the impact on the labor force's total employment and employment structure, the analysis of AI on employment quality should also be considered. Hui and Jiang (2023) found that AI technological advances can improve labor compensation. As the level of digital governance increases, the greater the improvement of employment quality. Qi and Tao (2023) used the dimensions of labor compensation, job stability and intensity, and social security to comprehensively measure the quality of employment and study the impact of industrial intelligence on the quality of employment of migrant workers. The study found that the employment quality of low-skilled migrant workers is more seriously affected by industrial intelligence. Acemoglu and Restrepo (2018) argued that the use of industrial robots has an impact on the wage level of the labor force, and the addition of one industrial robot per 1,000 workers will make the wage drop by 0.25–0.5 percentage points. Graetz and Michaels (2018) argued that technological progress has an impact on the total and structural effects of labor force employment while at the same time having an upward effect on labor force wage levels in all industries.

In summary, the impact of AI technological progress on labor force employment patterns has achieved certain results, but there are still two overall shortcomings. First, foreign research is mostly based on developed countries, whose economic development model and industrialization process differ from their own national conditions. Moreover, developing countries, as an important target for promoting AI and digital transformation, have relatively little research on it. Second, domestic quantitative research on the relationship between AI and labor force employment is also gradually emerging, but the research results are not uniform, and more data and facts are still needed to support the evidence. Therefore, this study explores the mechanism and empirical analysis of the impact of AI development on the employment pattern of the manufacturing labor force to provide evidence for the research on this issue.

## 3 Mechanistic analyses and research hypotheses

The application of AI in the manufacturing industry has attracted a lot of attention, and the mechanism analysis and research hypotheses about it have been proposed around the employment patterns of the labor force in the three aspects of total employment, employment structure, and employment quality.

### 3.1 The impact of AI on total employment in manufacturing

At this stage, AI technology represented by industrial robots is gradually gaining popularity. Intelligence and automation have been applied in life. AI is applied in many production processes such as product development, production and packaging, quality control, and logistics and distribution, and at the same time, it has the development advantages of high precision, high speed, flexibility, and safety, which comprehensively improves the production efficiency of enterprises, reduces the operating costs, enhances the controllability of the product life cycle, and produces both direct and indirect substitution effects on labor force employment (Wang and Dong, 2020). The direct substitution effect is manifested in the automated production line robots, which may replace some traditional, highly repetitive, manual labor-based work. The indirect substitution effect is manifested in the fact that with the introduction of intelligent equipment, the productivity of the unit product is increased while the labor demand is reduced.

At the same time, AI creates new types of jobs for the labor force (Yang et al., 2018). AI can replace the simpler labor force but not the complex needs of the job. For example, intelligent customer service is still unable to provide high-quality services equivalent to human customer service at this stage. The development of AI promotes the development, operation, and maintenance of intelligent products. In the process of intelligence, engineers engaged in developing new technologies are required to be responsible for technological upgrades, professional data analysts are needed to handle huge amounts of data, and senior technicians are needed for system integration, debugging, and maintenance. The application of AI covers a wide range of professional skills, from hardware to software, providing more development opportunities for high-skilled practitioners in the technology and data fields.

Thus, the impact of AI on total employment in manufacturing is inextricably linked to the substitution and the creation effects. In the short term, the substitution effect dominates. The introduction of automation technologies may lead to the replacement of some traditional and highly repetitive jobs, thus negatively affecting total employment. In the long run, the creation effect plays a dominant role. The development of new technologies may give rise to new industries and may bring about complex and high-value-added jobs that require more high-skilled people to add to the mix, further promoting job creation. This suggests the following hypothesis:

Hypothesis 1: AI has a positive *U*-shaped relationship with total manufacturing employment, with the short-term dominated by the substitution effect and the long-term dominated by the creation effect, and the total employment decreases first and then rises.

### 3.2 The impact of AI on the employment structure in manufacturing

The impact of AI on manufacturing employment covers both labor forces at different skill levels (Sun and Hou, 2021). The high-skilled labor force is mainly responsible for developing intelligent products using machine learning, deep learning, and natural language processing, maintaining automated equipment, carrying out data mining, and formulating decision support systems, which play a key role in promoting manufacturing intelligence and improving productivity and product innovation. For the middle-skilled labor force, on the one hand, some simple jobs may be replaced by the introduction of automation equipment. Enterprises spend significantly more on hiring middle-skilled labor than low-skilled labor, which will more likely reduce the demand for middle-skilled labor. On the other hand, middle-skilled labor may be trained to grow rapidly into high-skilled labor. For low-skilled labor, the introduction of automated devices may lead to a reduction in the number of low-skilled jobs, with a higher probability of being replaced. At the same time, some of the low-skilled jobs may come from the complement of the middle-skilled labor force. The low-skilled labor force grows as the middle-skilled labor force is unable to adapt to technological advances and has to choose to move to a lower level of employment. Therefore, the impact of AI on the labor force of different skills needs to be considered as a combination of substitution and creation effects. For the lower-skilled labor force, it is necessary to focus on training and transformation and continuously learn and update their skills to cope with the ever-changing demands of the manufacturing industry.

Hypothesis 2: As the level of AI development increases, low-skilled labor is more likely to be replaced.

### 3.3 The impact of AI on employment quality in manufacturing

Technological advances in AI can help improve manufacturing productivity, improve the working environment, and create job opportunities while increasing the demand for skills training and capacity enhancement among the labor force, which has a positive and active impact on the quality of employment. The application of AI technology can optimize the production process, create more business opportunities for enterprises, and increase the output value of workers' units. It can provide more high-quality jobs for workers and make the employment structure more diversified. It can enhance the creativity of workers. In addition, it can carry out some high-risk work tasks, but personal safety can be safeguarded at the same time.

Income serves as a key indicator of the quality of manufacturing employment. It is itself formed by pooling a variety of goods and services in market transactions, reflecting a number of aspects such as labor compensation, social security and welfare, job stability, and wage gap. Katz and Murphy (1992) argued that the impact of technological progress on income distribution is biased. The level of development of AI makes differences in the income level of the labor force by affecting the total employment and the employment structure of the labor force, which in turn triggers the turnover of old and new jobs and changes in the share of labor income (Wang et al., 2020). In the development process of urban–rural integration, AI promotes the transformation of traditional manufacturing to digital manufacturing, vigorously promotes industrial upgrading, and improves industrial added value. In e-commerce and digital platforms, AI provides a broader market for small and micro enterprises, makes it easier for farmers to access information, education, and resources, and helps small-scale manufacturing enterprises in rural areas to better participate in the global supply chain. In the agricultural sector, AI technology is widely used to develop agricultural mechanization, improving the efficiency of agricultural production and increasing farmers' income. From this, the following is proposed:

Hypothesis 3: AI is instrumental in the overall improvement of employment quality in the manufacturing sector and the easing of the urban–rural income gap.

## 4 Theoretical modeling and research design

### 4.1 Theoretical modeling

Task models, originally proposed by Zeira (1998) and later extended by Acemoglu and Autor (2011), are used to analyze a variety of issues, such as the impact of AI on economic growth and the labor market. Assume that the production process is carried out through a series of tasks, denote the sectoral level of production by *Y*, and *Y*(τ) denotes the output of task τ, taking the range τ ∈ [*N* − 1, *N*]. σ is the inter-task elasticity of substitution, which refers to the substitution between the factors of labor and takes the form of Equation (1):


(1) $$\begin{array}{l} \begin{array}{c} Y={\left(\int_{N-1}^{N}Y{\left(\tau \right)}^{\frac{\sigma -1}{\sigma }}d\tau \right)}^{\frac{\sigma }{\sigma -1}} \end{array} \end{array}$$

Equation (1) describes the impact of factors of production on the configuration of the task model and automation and new tasks. Taking a single sectoral economic production process as an example, it contains both capital and labor production when τ ∈ [*N* − 1, *I*], and only labor production when τ ∈ (*I, N*). An increase in *I* indicates the introduction of automation technology, and an increase in *N* indicates the introduction of new labor-intensive tasks. In addition to automation and introducing new tasks, the sectoral technology profile depends on labor-augmenting (*A*^*L*^) and capital-augmenting (*A*^*K*^) technologies.

Based on the production function in Equation (1), the output *Y*(τ) of task τ can be further expressed as:


(2) $$\begin{array}{l} Y\left(\tau \right)=\{\begin{array}{c} \begin{array}{c} A^{L}\gamma^{L}\left(\tau \right)l\left(\tau \right)+A^{K}\gamma^{K}\left(\tau \right)k\left(\tau \right)\tau \in \left[N-1,I\right] \end{array}\\ \begin{array}{c} A^{L}\gamma^{L}\left(\tau \right)l\left(\tau \right)\tau \in \left(I,N\right] \end{array} \end{array} \end{array}$$

where $L=\int_{N-1}^{N}l\left(\tau \right)d\tau$ and $K=\int_{N-1}^{N}k\left(\tau \right)d\tau$ denote labor and capital in production, respectively, and this expression also shows how automation and new tasks can affect the task model allocation. AI can both increase the scope of automation and create new jobs, and the impact on the production level in each industry is related to the relative size of the substitution and creation effects.

Next, by substituting Equation (2) into Equation (1), the Constant Elasticity of Substitution (CES) is defined as:


(3) $$\begin{array}{l} Y=({\left(\int_{N-1}^{I}\gamma^{K}{\left(\tau \right)}^{\sigma -1}d\tau \right)}^{\frac{1}{\sigma }}{\left(A^{K}K\right)}^{\frac{\sigma -1}{\sigma }}\\ \;{+{\left(\int_{I}^{N}\gamma^{L}{\left(\tau \right)}^{\sigma -1}d\tau \right)}^{\frac{1}{\sigma }}{\left(A^{L}L\right)}^{\frac{\sigma -1}{\sigma }})}^{\frac{\sigma }{\sigma -1}} \end{array}$$

Consider that the labor share parameter Γ(*I, N*) and the total factor productivity Π(*I, N*) can be expressed as Equation (4):


(4) $$\begin{array}{l} \begin{array}{c} \Gamma \left(I,N\right)=\frac{\int_{I}^{N}\gamma^{L}{\left(\tau \right)}^{\sigma -1}}{\int_{N-1}^{I}\gamma^{K}{\left(\tau \right)}^{\sigma -1}d\tau +\int_{I}^{N}\gamma^{L}{\left(\tau \right)}^{\sigma -1}}\;,\\ \Pi \left(I,N\right)={\left(\int_{N-1}^{I}\gamma^{K}{\left(\tau \right)}^{\sigma -1}d\tau +\int_{I}^{N}\gamma^{L}{\left(\tau \right)}^{\sigma -1}\right)}^{\frac{1}{\sigma -1}}\; \end{array} \end{array}$$

Further, based on Equation (4), Equation (3) is deformed and the CES production function can be expressed as Equation (5):


(5) $$\begin{array}{l} \begin{array}{c} Y=\Pi \left(I,N\right)(\Gamma {\left(I,N\right)}^{\frac{1}{\sigma }}{\left(A^{L}L\right)}^{\frac{\sigma -1}{\sigma }}\\ \;+{{\left(1-\Gamma \left(I,N\right)\right)}^{\frac{1}{\sigma }}{\left(A^{K}K\right)}^{\frac{\sigma -1}{\sigma }})}^{\frac{\sigma }{\sigma -1}} \end{array} \end{array}$$

Overall, AI can substitute or create labor employment and change the capital–labor ratio. That is, AI has a substitution or creation effect on labor employment. In the above production function, *I* is introduced as an automation technology, further affecting Π(*I, N*) and Γ(*I, N*). At this stage, changing the proportions of the task model that are controlled by capital or labor allows the level of employment development at equilibrium to change.

### 4.2 Data sources and variable descriptions

The data that come from the China Statistical Yearbook, China Labor Statistical Yearbook, and China Population and Employment Statistical Yearbook are calculated and aggregated based on publicly available data from relevant departments. The panel data of 31 provinces and cities in China for 11 years from 2011 to 2020 is used to study the impact of the development of AI on total employment, employment structure, and employment quality of the manufacturing labor force. The descriptions and illustrations of the specific indicator variables are shown in Table 1.

**Table 1:** Description and illustration of indicator variables.

**Variable type**	**Variable name**			**Sign**	**Unit**	**Data sources and instruction**	
Dependent variable	Total employment		Number of manufacturing employment	L	Million people	China statistical yearbook	
	Employment structure	Proportion of skilled workers in manufacturing	Low-skilled	Low	%	China Labor Statistical Yearbook	Junior high school and below
			Middle-killed	*Middle*	%		Junior college or high school
			High-skilled	High	%		bachelor degree and above
		Number of employed persons in different industries		*y*_*i*_	Million people	China Statistical Yearbook	
	Employment quality	Urban-rural income gap		G	——	China Statistical Yearbook	Urban per capita disposable income/rural per capita net income
		Urban disposable income per capita		Yu	——	China Statistical Yearbook	
		Rural net income per capita			Yr				
Independent variable	The development level of AI			AI	——	Entropy method	
Control variable	Regional GDP per capita			Agdp	Yuan	China Statistical Yearbook	
	Manufacturing fixed assets investment of the whole society			Pap	Billion yuan		
	The proportion of labor population in the manufacturing industry			Fai	——		
	The average wage of manufacturing employees			Wage	Million yuan		
	urbanization level			Urban	——	China Population and Employment Statistics Yearbook	Urban population/total population
	export			Export	thousand US dollars	Total export volume of the place where the business unit is located	

The data used in this study are logarithmically processed based on the original variables of the table.

#### 4.2.1 Dependent variables

When analyzing total employment, the logarithm of the number of employed persons in the manufacturing industry is chosen as the dependent variable to compare the size of the substitution effect and the creation effect. When analyzing the employment structure, the logarithm of the ratio of employed persons with different skills in the manufacturing industry is chosen as the dependent variable to explore the effect of the polarization of heterogeneous labor force employment. Since the number of employed persons with different skills in the manufacturing industry cannot be obtained directly, this study calculates the proportion of employed persons with different skills based on the Composition of Educational Levels of Employed Persons in Sub-regions of the Country in China Labor and Employment Yearbook and then calculates the number of persons in each region of the manufacturing industry based on the proportion of the manufacturing industry as a whole and the number of persons working in the whole industry, and then obtains the number of employed persons with different skills by multiplying the two together. At the same time, based on the input–output table, industries with greater forward and backward linkages with the manufacturing industry were selected to analyze the inter-industry spillover effect. When analyzing the quality of employment, we chose the ratio of disposable income per capita of urban residents to net income per capita of rural residents to measure the urban–rural income gap and analyzed the logarithms of disposable income per capita of urban residents and net income per capita of rural residents as dependent variables, respectively, and the logarithm of disposable income per capita of urban residents and the logarithm of net income per capita of rural residents are analyzed as dependent variables, respectively.

#### 4.2.2 Independent variable

Considering human error, the entropy value method is now used to determine the indicator weights according to the degree of variation of each sub-indicator and then measure the level of AI development. Among them, four categories of sub-indicators are selected, namely, the full-time equivalent of R&D personnel of industrial enterprises above large scale (person/year), the application of industrial robots (US $ million/person), the ratio of fixed assets of the whole society to the GDP of the information transmission, computer services, and software industry, and the productivity of AI technology. First, due to the lack of data on industrial robots in each province, and taking into account that more than 70% of industrial robots rely on imports and are mainly in the secondary industry, the ratio of the output value of the secondary industry in each province is taken as the weight, and the amount of imports of industrial robots is allocated to the province, and then to the people. The specific calculation formula is as follows: the use of industrial robots is calculated by multiplying the number of imported industrial robots and the share of the region's secondary sector output in GDP, and then dividing the calculated value by the number of permanent residents at the end of the year (Chen and Hu, 2020). Second, the productivity of AI technology is equal to the inverse of the price index of investment in fixed assets. Third, the fixed asset investment price index for the Tibet region has not been published, so we are now drawing on the practice of Shan (2008) in Quantitative Economic and Technical Economic Research to fill in this missing value using the price indices for Xinjiang and Qinghai provinces (Shan, 2008).

Taking the total employment as an example, based on the research on the theoretical mechanism of the task model, after comparing the fixed effect and random effect models, it is determined that the fixed effect model is better and passes the Hausman test. To further eliminate the influence of the quantitative scale, the selected variables are logarithmically processed, where *L* is the number of employment in the manufacturing industry, *AI* represents the level of development of AI, the control variable *C* is added at the same time, β_0_ is the intercept term, β_1_, β_2_ are the coefficients of the influence of the level of development of AI on the manufacturing industry's labor inputs, and μ_*it*_ is the random error term, and the specific expressions are as follows:


(6) $$\begin{array}{l} {\ln\;L}_{it}=\beta_{0}+\beta_{1}{\ln\;AI}_{it}+\beta_{2}\ln\;C+\mu_{it} \end{array}$$

Combining the non-linear relationship between the substitution and creation effects in total employment and the level of AI development, the squared term of the level of AI development is included in the equation for consideration. β_3_ is the degree of influence of each control variable. The expression is as follows:


(7) $$\begin{array}{l} \begin{array}{c} \begin{array}{c} {\ln\;L}_{it}=\beta_{0}+\beta_{1}{\ln\;AI}_{it}+\beta_{2}{\ln\left(AI_{it}\right)}^{2}+\beta_{3}\ln\;C \end{array}+\mu_{it} \end{array} \end{array}$$

For studies that analyze employment structure and employment quality with the level of AI development, it is only necessary to replace the labor input *L* in Equations (6) or (7) with the corresponding explanatory variables.

## 5 Empirical analysis

### 5.1 Benchmark regressions

Considering the study is on panel data, the benchmark regression adopts the fixed effects model. After the Hausman test, there is heteroskedasticity and serial correlation in the model, which needs to be further corrected by Feasible Generalized Least Squares (FGLS), and the specific results of the benchmark regression are shown in Table 2, with the province and year fixed in all the empirical processes.

**Table 2:** Benchmark regression.

**Variable**	**Total employment**		**Employment structure**			**Employment quality**		
	**No** ***ln (Ai)**^2^*	**With** ***ln (Ai)**^2^*	***ln low***	***ln middle***	***ln high***	***ln G***	***ln Yu***	***ln Yr***
	**(1)**	**(2)**	**(3)**	**(4)**	**(5)**	**(6)**	**(7)**	**(8)**
*ln Ai*	−0.072^*^	−0.170^***^	−0.261^***^	−0.160^***^	−0.165^**^	−0.109^***^	0.015^**^	0.069^***^
	(0.040)	(0.057)	(0.064)	(0.093)	(0.062)	(0.013)	(0.006)	(0.016)
*ln (Ai)^2^*		0.088^***^	0.040	−0.023	−0.047			
		(0.026)	(0.418)	(0.051)	(0.047)			
Control variable	Yes	Yes	Yes	Yes	Yes	Yes	Yes	Yes
Provincial fixed effect	Yes	Yes	Yes	Yes	Yes	Yes	Yes	Yes
Year fixed effect	Yes	Yes	Yes	Yes	Yes	Yes	Yes	Yes
_cons	0.454	0.386	−84.451^***^	−10.540	43.840	31.256^***^	−0.234	−107.949^***^
	(1.570)	(1.231)	(23.230)	(19.764)	28.471	(8.699)	(0.347)	(10.458)
*R* ^2^	0.920	0.997	0.873	0.858	0.869	0.608	0.994	0.946

Standard errors in parentheses, ^*^p < 0.1, ^**^p < 0.05, ^***^p < 0.01, below.

For total employment, the difference between columns (1) and (2) is whether the squared terms of the explanatory variables are included. Observing the coefficient values of the explanatory variables, the values of the coefficients of the explanatory variables in column (1) are significantly negative at the 5% level. The number of manufacturing employees decreases as the level of AI development increases, and the employment substitution effect is greater than the creation effect. In column (2), after adding the square term, the coefficient sign is the opposite, and according to the coefficients of the primary and secondary terms in the regression model, the level of development of AI on the total number of employment impact first declined and then increased, showing a positive *U*-shaped relationship change. It suggests that the level of AI development is dominated by a substitution effect on total employment in the early stages and a creation effect in the later stages. Take column (2) as an example; when the level of AI development is less than the inflection point of 2.57,^1^ the substitution effect is greater than the creation effect and the number of employed people decreases. When the level of AI development is >2.57, the creation effect is greater than the substitution effect, new jobs increase, and total employment increases. Overall, there is a non-linear relationship between AI and total employment, and the substitution effect is greater than the creation effect in the short term, more skilled labor is replaced, and the supply of skilled personnel cannot be met. In the medium and long term, the creation effect is greater than the substitution effect, more jobs will be created, and more skilled labor needs to be absorbed to promote balanced economic development. Hypothesis 1 is valid.

In terms of employment structure, the employed persons are divided into low-skilled group (junior high school and below), middle-skilled group (college or high school), and high-skilled group (bachelor's degree and above) based on their education level, which is used to analyze the changes in the level of AI development of the employed persons with different skills. Since the substitution effect still dominates at present, and considering the stability of work and income volatility, the square term of the explanatory variables is not added, and when studying the analysis of employment structure and employment quality, only the coefficient changes of the primary term of the explanatory variables are analyzed, and the results are shown in columns (3), (4), and (5) in Table 2. From the coefficient values, every 1% increase in the level of AI development results in a different degree of reduction in the number of employed persons of all skills, and the degree of reduction becomes smaller with the increase in the level of skills, i.e., the assertion in Hypothesis 2 about the “greater likelihood of low-skilled labor force substitution” is confirmed.

For the employment quality, the urban–rural income gap, the logarithm of disposable income per capita for urban residents, and net income per capita for rural residents are regressed sequentially as explanatory variables, as shown in columns (6), (7), and (8). Considering the high-frequency changes in income, the squared terms of the explanatory variables are not included, and only the short-term effects are analyzed. Column (6) shows that as the level of AI development increases, the inputs of intelligence and mechanization lead to a significant reduction in the urban–rural income gap and the easing of social conflicts. Columns (7) and (8) further analyze the extent to which the income levels of urban and rural residents are affected by the development of AI, and the increase in the level of AI development is accompanied by an increase in the per capita disposable income of urban residents and the per capita net income of rural residents, and the extent to which rural residents are affected is greater than that of urban residents, and the overall development is positive. Considering that land is the main source of income for farmers, with the introduction of intelligent agricultural equipment, the quality of farm seeds, and planting concepts, the level of income increases. As for urban residents, who rely mainly on their jobs for income, technological progress has had an impact on urban employment but has not brought about serious employment difficulties, and the income level of urban residents has also risen slightly. Thus, Hypothesis 3 is valid.

### 5.2 Analysis of regional heterogeneity

To further study the heterogeneity of AI on inter-regional manufacturing employment, referring to Yang et al. (2015), *m* is set as a regional dummy variable, such as when studying the East Coastal integrated economic zone, the dummy variable *m* = 1 is set, and the other integrated economic zones are set to *m* = *0*. Considering that both the explanatory variables and the explanatory variables are continuous variables and the moderating variables are categorical variables, the grouped regression is the chosen method, and the specific results are shown in Table 3; regression results for explanatory variables are presented.

**Table 3:** Interregional grouping regressions.

**Variable**		**North East**	**North Coastal**	**East Coastal**	**South Coastal**	**Yellow River Middle Reach**	**Yangtze River Middle Reach**	**Great Southwest**	**Great Northwest**
Total employment	*ln Ai*	−0.613^**^	−1.648^***^	0.562	−0.512^***^	−0.525^**^	1.417^***^	−0.077^***^	−0.096
		(0.278)	(0.611)	(0.598)	(0.190)	(0.205)	(0.421)	(0.025)	(0.107)
	*ln (Ai)^2^*	0.320^**^	0.952^***^	−0.111	0.402^***^	0.182	−0.706^***^	0.069	−0.000
		(0.140)	(0.252)	(0.174)	(0.104)	(0.112)	(0.231)	(0.045)	(0.063)
Employment structure *lnAi*	*ln low*	−0.246	−0.009	0.084	−0.333^**^	−0.592^***^	0.797^***^	−0.025	−0.086
		(0.533)	(0.381)	(0.526)	(0.139)	(0.194)	(0.198)	(0.024)	(0.107)
	*ln middle*	−0.647^*^	−1.128^***^	1.694^***^	−0.430^***^	−0.362^**^	0.716	0.020	−0.098
		(0.396)	(0.410)	(0.437)	(0.137)	(0.175)	(0.531)	(0.026)	(0.133)
	*ln high*	−0.975	−0.818	1.959^***^	−0.359^**^	−0.750^**^	0.449	0.076	−0.006
		(0.634)	(0.634)	(0.645)	(0.159)	(0.312)	(0.788)	(0.048)	(0.108)
Employment quality, *lnAi*	*ln G*	−0.032^*^	−0.000^*^	−0.069^*^	0.022^*^	−0.017^*^	−0.020	0.031^**^	0.012^**^
		(0.018)	(0.042)	(0.039)	(0.011)	(0.010)	(0.014)	(0.013)	(0.006)
	*ln Yu*	−0.048^*^	0.099^***^	0.049	0.051^***^	−0.044^*^	0.095^***^	0.018^*^	0.011^**^
		(0.026)	(0.037)	(0.066)	(0.014)	(0.025)	(0.027)	(0.010)	(0.005)
	*ln Yr*	−0.063^***^	0.098^***^	0.017	0.047^*^	0.016	0.126^***^	0.084^***^	0.112^**^
		(0.023)	(0.022)	(0.043)	(0.027)	(0.030)	(0.015)	(0.029)	(0.044)

Standard errors in parentheses, ^*^p < 0.1, ^**^p < 0.05, ^***^p < 0.01.

In terms of total employment, except for the East Coast Comprehensive Economic Zone and the Great Northwest Comprehensive Economic Zone, the impact of AI on employment in most regions is significant. It is noteworthy that the primary term coefficient of the Yangtze River Middle Reach Comprehensive Economic Zone is positive, and the secondary term coefficient is negative, indicating an inverted *U*-shaped relationship in the region. When the level of AI development is <2.72, the creation effect dominates. When it is >2.72, the substitution effect occupies a major position, and manufacturing employment decreases. As for the comprehensive economic zone in the Yangtze River Middle Reach, as mentioned by the National Development and Reform Commission in the “14th Five-Year Plan for the Development of Urban Agglomeration in the Yangtze River Middle Reach”, although the comprehensive strength and development capacity of the central city in the region are constantly improving, the radiation drive is not strong enough, the ability to transform key technologies and innovations is insufficient, and there are fewer industries with core competitiveness. There are fewer industries with core competitiveness. At the beginning of the AI technology launch, in the short term, multiple industry types developed together, the demand for skilled personnel increased and was inclusive, and more jobs were offered. However, as the level of AI development rises, the competitive pressure on the labor force is greater, posing a threat to the less skilled labor force, and the substitution effect continues to dominate. The rest of the significant regional employment is consistent with the overall positive U-shaped development relationship. The substitution effect is greater than the creation effect in the early stage, and the creation effect is greater than the substitution effect in the later stage.

In terms of the employment structure, the main comparison is between 2011 and 2020 in terms of the number and share of employed persons in the regions with different skill components (see Table 4). Compared with 2011, first, high-skilled employed persons have all risen to different degrees. There is a complementary relationship between technological development and high-skilled labor, with the introduction of high-skilled personnel driving up productivity. The Great Southwest Comprehensive Economic Zone saw the largest rise in the number of personnel, followed by the South Coast and East Coast Comprehensive Economic Zones. The North Coastal Comprehensive Economic Zone has the highest proportion of high-skilled personnel, but the region has the lowest rise in high-skilled personnel and had high-skilled personnel that surpassed the rest of the region in 2011, suggesting that its growth is now bottlenecked at this stage. Second, for middle-skilled employment, the number of personnel in the Northeast Comprehensive Economic Zone and North Coastal Comprehensive Economic Zone has decreased, and the proportion of middle-skilled personnel in the North Coastal Comprehensive Economic Zone has declined and the rest of the region has risen. The rate of increase year-on-year was lower than that of high-skilled employment, and the most significant rise was still in the Greater Southwest Comprehensive Economic Zone, where the short-term imbalance between labor supply and demand caused by the “machine replacement” is still being felt. Third, for low-skilled employment, headcounts declined in most regions, with the largest decline occurring in the Northeast integrated economic region, which is less inclusive of both low-skilled and middle-skilled workers. Only the South Coast and the Greater Southwest Integrated Economic Zones rose slightly, but the change was not significant. Low-skilled workers have been hit harder by technological advances, even to the point of losing their jobs, and the downward shift of some of the middle-skilled workers will further increase the share of low-skilled workers. Taken together, the Greater Southwest Integrated Economic Region has a stronger demand for middle-skilled and high-skilled personnel, and the Northeast Integrated Economic Region has a weaker inclusiveness for low-skilled and middle-skilled personnel. Combined with the first-order grouping regression results for each regional skill set in Table 3, the regions are further classified into four categories. The first category is the Great Southwest and the Great Northwest Comprehensive Economic Zones, where there is more room for improvement in the level of AI development, and there is demand for personnel with different skills. The second category is the Northeast, Northern Coastal, and Middle Reaches of the Yangtze River Integrated Economic Zones (ICZs), with the Northeast ICZ and Northern Coastal ICZ having a negative impact on middle-skilled employment, i.e., the impact of AI in the region on low-skilled and high-skilled is weak. The Yangtze River Middle Reach Comprehensive Economic Zone has a boosting effect on low-skilled employed persons, and the impact on middle-skilled and high-skilled is not significant. It shows that the economic development level of this type of region is low, and it also needs to be assisted by the promotion of high-skilled personnel and low-skilled personnel, and it is more concentrated in labor-intensive industries. The third category is the Southern Coastal and the Yellow River Middle Reach Comprehensive Economic Zone, and the two regions have a different degree of reduction of the employed personnel of different skill groups, which, in combination with the level of the development of AI in this type of region in recent years, shows that the level of its intelligent development is higher, and the substitution effect occupies the dominant position. The fourth category is the Eastern Coastal Comprehensive Economic Zone, which has the highest level of AI development, a pulling effect on the employment of middle-skilled and high-skilled personnel, strong inclusiveness, rapid development, strong creation effect, and an increase in the number of new jobs.

**Table 4:** Interregional employment status by skill group.

**Area**	**Year**	**Low-skilled**		**Middle-skilled**		**High-skilled**	
		**Numbers**	**Proportion**	**Numbers**	**Proportion**	**Numbers**	**Proportion**
North East	2011	240.031	0.769	63.549	0.204	8.542	0.027
	2020	139.088	0.700	47.573	0.239	12.042	0.061
North Coastal	2011	342.331	0.592	194.475	0.336	41.615	0.072
	2020	267.776	0.571	146.950	0.313	54.573	0.116
East Coastal	2011	344.725	0.654	158.005	0.300	24.773	0.047
	2020	303.036	0.566	177.230	0.331	54.742	0.102
South Coastal	2011	301.963	0.667	137.213	0.303	13.203	0.029
	2020	312.882	0.598	178.910	0.342	31.469	0.060
Yellow River Middle Reach	2011	326.695	0.721	116.107	0.256	10.244	0.023
	2020	268.067	0.656	119.661	0.293	21.058	0.052
Yangtze River Middle Reach	2011	315.668	0.713	116.202	0.263	10.734	0.024
	2020	283.436	0.672	117.826	0.280	20.267	0.048
Great Southwest	2011	487.747	0.730	166.122	0.248	14.696	0.022
	2020	490.041	0.644	231.414	0.304	40.016	0.053
Great Northwest	2011	100.946	0.726	33.919	0.244	4.173	0.030
	2020	93.520	0.681	35.671	0.260	8.139	0.059

Number of units: ten thousand people.

In terms of employment quality, as the level of AI development increases, the urban–rural income gap decreases in the Northeast, East Coast, and Middle Yellow River Comprehensive Economic Zones and increases in the South Coast, Great Southwest, and Great Northwest Comprehensive Economic Zones; however, the urban–rural income gap in the North Coast and Yangtze River Middle Reach Comprehensive Economic Zones is not significant, which suggests that technological upgrading does not necessarily bring about a positive response in all regions and that its response is not necessarily a function of regional comprehensive development. The significance and direction of the response are inseparable from the comprehensive development of the region. Specifically, on the one hand, the disposable income per capita in urban areas and net income per capita in rural areas in the North Coastal, South Coastal, Yangtze River Middle Reach, Great Southwest, and Great Northwest Comprehensive Economic Zones are positively proportional to the level of AI development, and both urban and rural incomes have increased in the wake of technological upgrading. However, among them, the urban–rural income gap is gradually widening while the income level of the Southern Coastal, Great Southwest, and Great Northwest Comprehensive Economic Zones is improving, indicating that the overall improvement of urban residents in the above regions is higher than that of rural residents. On the other hand, the urban per capita disposable income and rural per capita net income of the Northeast Comprehensive Economic Zone are inversely proportional to the level of AI development. The urban and rural residents' incomes are both decreasing at the same time of technological upgrading, but the urban–rural income gap, however, has decreased, indicating that the overall decline of urban residents is higher than that of rural residents, and the region's economic trend is downward.

### 5.3 Industry spillover effects

Considering the large linkage between the manufacturing industry and its upstream and downstream related industries, it is necessary to further analyze the spillover effect of AI on the upstream and downstream related industries of the manufacturing industry, and it is especially necessary to explore the mobility of the employment group to the related industries outside the manufacturing industry. Combined with the 2020 input–output table, the direct consumption coefficient between industries is calculated, and the forward linkage effect and backward linkage effect are calculated. Among them, the forward (backward) linkage effect refers to the changes in production, output value, technology, and other aspects of an industry that cause changes in the corresponding aspects of its forward (backward) related sectors.


(8) $$\begin{array}{l} \begin{array}{c} Lf\left(i\right)=\frac{\sum X_{ij}}{X_{i}}\; \end{array}\left(i=1,2,\ldots ,n\right),\\ Lb\left(i\right)=\frac{\sum X_{ij}}{X_{j}}\;\left(j=1,2,\ldots ,n\right) \end{array}$$

Where *Lf*(*i*) denotes the forward correlation effect of upstream industries and *Lb*(*j*) denotes the backward correlation effect of downstream industries. *i* and *j* are different industrial sectors, and *X* is the intermediate input matrix. Based on Equation (8), combined with the 2020 input–output table and the industry division guidelines of the China Statistical Yearbook, the top five industries in the direct consumption coefficients of the upstream and downstream industries are selected, respectively. Furthermore, setting the logarithm of the number of employed persons in the top five corresponding industries in terms of industry linkage as the explanatory variable, the control variables remain unchanged, and no squared term is added to analyze the manufacturing employment association effect according to the absolute size of the coefficients. The results are shown in Table 5.

**Table 5:** Industry linkages.

**Upstream industries**	**Backward linkage**	**Variable**	**β_1_**	**Downstream industries**	**Forward linkage**	**Variable**	**β_1_**
Construction	0.748	y1	−0.366^***^	Mining	1.518	y6	−0.660^***^
Electricity, heat, gas, and water production	0.685	y2	−0.213^***^	Rental and business services	0.929	y3	−0.303^***^
Rental and business services	0.669	y3	−0.303^***^	Electricity, heat, gas and water production	0.855	y2	−0.213^***^
Accommodation and catering	0.663	y4	−0.179^**^	Transport, storage and postal services	0.772	y7	−0.259^***^
Scientific research and technical services	0.626	y5	−0.224^***^	Finance	0.722	y8	−0.123^***^

Standard errors in parentheses, ^*^p < 0.1, ^**^p < 0.05, ^***^p < 0.01.

The upstream and downstream industries of the manufacturing industry should have a mutually beneficial and co-existing relationship. Among the upstream industries, the one most affected by the manufacturing industry is the construction industry; among the downstream industries, the one most affected by the manufacturing industry is the mining industry. It is worth noting that the direct consumption coefficients of the two industries, namely, electricity, heat, gas, and water production and leasing and business services, are at a high level in both the upstream and downstream aspects, indicating that these two industries are more affected by the manufacturing industry. In comparison, the manufacturing industry has more influence on the downstream industries of these two industries. At the same time, the correlation effect of the manufacturing industry on the above industries is negative and significant in all industries, indicating that the impact of AI on the labor market is mainly dominated by the substitution effect. For the creation effect formed by the expansion of production scale, upgrading of industrial structure and improvement of consumption structure has not yet been formed. From the point of view of the absolute size of the coefficient, the manufacturing industry has the smallest degree of influence on the financial industry, followed by accommodation and catering. To a certain extent, this also shows that with the improvement of the level of development of AI, although the overall efficiency is improved, some machines that cannot replace human work still exist, and it is not easy to replace.

### 5.4 Robustness and endogeneity tests

To further verify the robustness of the empirical results, robustness tests are conducted for each part of the benchmark regression.

(1) Divide the sample period. Considering the sample span problem, the existing sample is divided into two periods, 2011–2015 and 2016–2020. The influence of the development level of AI on the number of employees in the manufacturing industry is measured in stages, and the results are shown in columns (1) and (2). The results show that, within both phases, AI has a *U*-shaped correlation with the number of people employed in manufacturing, with negative first-order coefficients and positive second-order coefficients. The substitution effect dominates in the short run and the creation effect dominates in the long run, consistent with the results of the benchmark regression on total employment in Table 2.

(2) Replacement of explanatory variables. Estimation measures on the level of AI development can be roughly divided into two categories. One category is the comprehensive evaluation by constructing an indicator system, such as the estimation using the entropy value method, as we did in this study. The other category can be reflected by using robot data, including robot penetration rate and number of robots. Therefore, the robot installation density indicator from the International Federation of Robotics data is used here as an explanatory variable for the overall regression analysis validation. Column (3) is the benchmark regression on total employment, with negative first-order coefficients and positive second-order coefficients, showing a positive *U*-shaped curve. Consistent with the measurements that divide the sample period, the same confirms the robustness of the benchmark regressions of total employment in Table 2. Columns (4), (5), and (6) are benchmark regressions on the structure of employment in different skill groups, with positive *U*-shaped curves for both employment and AI development levels in different skill groups, consistent with the aggregate. There is a negative effect in the short run, with higher levels of development resulting in less demand for personnel and a greater likelihood of replacement, and a positive effect in the long run.

(3) Replacement of explanatory variables. Considering that the urban–rural income gap is more sensitive to changes in income polarization, the measure was replaced by the Thiel index from the original urban–rural income ratio. The formula for calculating the Thiel index: $T_{P}=\underset{i}{\sum }\underset{j}{\sum }\left(\frac{W_{ij}}{W_{i}}\right)\log\left(\frac{W_{ij}P_{ij}}{YP}\right)$, where *i* = 1, 2 represents towns and villages, respectively, *j* represents provinces, and *W* and *P* are income and population, respectively. The smaller the value of the Tel index, the smaller the income gap. The results of the measurement are shown in column (7) of Table 6, and the coefficient of its explanatory variable is calculated to be −0.099 and passes the significance test, which is consistent with the significance and direction of the benchmark regression of the urban–rural income gap in Table 2, indicating that the results of the measurement are robust.

**Table 6:** Robustness test.

**Variable**	**Dividing the sample period**			**Replace independent variables**			**Replace dependent variables**
	**Total employment**			**Employment structure**			**Employment quality**
	**2016–2020**	**2011–2015**	**The total**	**Low-skilled**	**Middle-skilled**	**High-skilled**	**Tyrell's index**
	**(1)**	**(2)**	**(3)**	**(4)**	**(5)**	**(6)**	**(7)**
*ln Ai*	−0.495^**^	−0.313^**^	−0.061^***^	−0.025^*^	−0.087^***^	−0.086^***^	−0.099^***^
	(0.111)	(0.110)	(0.025)	(0.015)	(0.022)	(0.030)	(0.017)
*ln (Ai)^2^*	0.213^***^	0.064^*^	0.099^**^	0.046^***^	0.054^***^	0.071^***^	
	(0.026)	(0.024)	(0.020)	(0.012)	(0.017)	(0.024)	
control variable	Yes	Yes	Yes	Yes	Yes	Yes	Yes
Provincial fixed effect	Yes	Yes	Yes	Yes	Yes	Yes	Yes
Year fixed effect	Yes	Yes	Yes	Yes	Yes	Yes	Yes
_cons	153.158^***^	108.754^**^	−2.285	−4.758	−0.301	2.319	−73.104^***^
	(14.817)	(37.558)	(1.494)	(0.779)	(1.306)	(1.817)	(13.132)
*R* ^2^	0.932	0.923	0.994	0.998	0.996	0.989	0.809

Standard errors in parentheses, ^*^p < 0.1, ^**^p < 0.05, ^***^p < 0.01.

The above analysis confirms that the level of AI development has a significant impact on the total employment, employment structure, and employment quality of the manufacturing labor force, but there may be a reverse causality between changes in labor force employment patterns and the level of AI development at the same time. For example, the increase in high-skilled employment will further enhance the level of AI development. Therefore, by analyzing the core explanatory variables followed by one period as endogenous variables, the two-stage least squares method is applied to test whether there is a problem of endogeneity in the study, and the specific results are shown in Table 7.

**Table 7:** Endogeneity test (IV regression).

**Dependent variable**	**Employees**			**Differences in income levels**			
**Independent variable**	**First stage**	**Second stage**		**First stage**		**Second stage**	
	***ln Ai***	***ln (Ai)** ^2^*	***ln labor***	***ln Ai***	***ln G***	***ln Yu***	***ln Y**_*r*_*
*ln Ai_1*	0.386^***^	0.039		0.684^***^			
	(0.096)	(0.151)		(0.046)			
*ln (Ai)^2^_1*	−0.050	0.244^**^					
	(−0.065)	(0.012)					
*ln Ai*			−0.244^**^		−0.078^***^	0.098^***^	0.098^***^
			(0.112)		(0.024)	(0.017)	(0.024)
*ln (Ai)^2^*			0.195^**^				
			(0.095)				
Control variable	Yes	Yes	Yes	Yes	Yes	Yes	Yes
Provincial fixed effect	Yes	Yes	Yes	Yes	Yes	Yes	Yes
Year fixed effect	Yes	Yes	Yes	Yes	Yes	Yes	Yes
_cons	−10.391^***^	−6.272	−2.831	−19.549	23.185^**^	−69.231^***^	−100.18^***^
	(3.745)	(5.896)	(−1.842)	(29.083)	(10.468)	(9.022)	(10.423)
*R* ^2^	0.879	0.942	0.994	0.834	0.588	0.921	0.939

Standard errors in parentheses, ^*^p < 0.1, ^**^p < 0.05, ^***^p < 0.01.

The first stage regression results are used to test whether the selected instrumental variables are correlated with the core explanatory variables. When the explanatory variable is employed persons, the relationship between *ln AI, ln (AI)*^2^ lagged one period and *ln AI, ln (AI)*^2^ is significantly positive at the 1% level with coefficient values of 0.386 and 0.244, respectively. When the explanatory variable is the difference in the level of income, the squared terms are not taken into account; the relationship between *ln AI* lagged one period and *ln AI* is significantly positive at the 1% level with a coefficient value of 0.684, and the F-statistics are all over 10, and there is no problem with weak instrumental variables. The second-stage regression results comparing the regression results in column (2) and columns (6), (7), and (8) in Table 2 show that the problem of endogeneity is not serious. The fact that the coefficient values change by a factor of 10 or less and the significance and direction remain consistent indicates that endogeneity is not serious, and the findings of the study are supported.

## 6 Conclusion and recommendations

Based on Acemoglu's task model, the study analyses the mechanism of the impact of AI on the employment pattern of the manufacturing industry. Meanwhile, using the provincial panel data of the manufacturing industry from 2011 to 2020 as a sample, we study the impact of AI development on the total employment, employment structure, and employment quality of China's manufacturing industry and analyze and validate the impact effect of AI on the labor force pattern of the manufacturing industry. The main conclusions are drawn: first, there is a positive *U*-shaped relationship between the level of AI development and the total manufacturing employment, and the development trend in most regions is consistent with the overall development. The substitution effect is higher than the creation effect in the short term. In the long term, the creation effect is higher than the substitution effect, the number of new jobs increases, and the labor force employment is relieved. The current development of AI in China is still dominated by the substitution effect on manufacturing labor employment. Second, the level of AI development has a negative effect on the employment of different-skilled labor in the manufacturing industry, and both the overall and regional heterogeneity analyses confirm that low-skilled employed people are more likely to be replaced. For industry spillover effects, among the top five upstream and downstream industries, the two industries of electricity, heat, gas, and water production and leasing and business services are upstream and downstream affected by the manufacturing industry, while the financial industry and accommodation and catering are relatively less affected by the manufacturing industry. Furthermore, the development of AI has improved the quality of employment in the manufacturing industry, which has a positive impact on the income level of urban and rural residents in general and in most regions, and the net income per capita of rural residents has increased to a stronger extent than the disposable income per capita of urban residents, which is conducive to the construction of urban–rural integration.

Based on the above conclusions, the following recommendations are made:

(1) Enhance the overall level of AI development in the manufacturing sector and play an employment-pulling role. ① Increase investment in fixed assets. Accelerate the production and application of AI equipment and take both hard and soft into account to achieve structural upgrading of the manufacturing industry in terms of AI and enhance the overall level of development; ② Encourage independent innovation. Give full play to the leading role of the experimental zone for the innovation and development of a new generation of AI, accelerate the cultivation of the digital economy, robotics, and other emerging industries of AI, and promote the upgrading of the industrial structure of manufacturing and other multi-industry industries; ③ Government departments actively take the lead in ensuring that the AI equipment is landed in conjunction with the enterprises to provide jobs, enhance the development of the vitality of the enterprises, and put the machines at the service of people to maximize the protection of the rights and interests of the workers.

(2) Attaching importance to skills training for the labor force and upgrading the quality of education. Focusing closely on the guiding ideology of the Opinions on Strengthening the Construction of High-skilled Personnel in the New Era, it has increased its efforts to cultivate high-skilled personnel. Focusing on major national strategies, major projects and key industries, and with market demand as the guide, give full play to the subjective initiative of the labor force and cultivate high-quality professional and complex talents.

(3) Strengthening the leading role of developed regions and supporting backward regions. ①Accelerate the construction of industrial parks in regions with innovation qualifications, establish pilot zones for training digital skills talents around the concept of digital China, upgrade the skills of the entire population, and create a high-tech AI industry chain in manufacturing; continue to strengthen the leading role of the Eastern Coastal Comprehensive Economic Zone in demonstrating the synergistic development of the manufacturing industry chain, and promote the organic integration of the upstream, midstream, and downstream industries; ② Strengthen the support of policies for remote regions and introduce intelligent project inputs; ③ Set up more attractive policies for the introduction of talents and high-quality enterprises and use high-quality resources to drive the development of regions in accordance with the development strategy of “bringing in and going out”. Strengthening policy support for remote areas, introducing intelligent project inputs, setting up more attractive policies for the introduction of talents and high-quality enterprises, and using high-quality resources to drive the development of the regional economy in accordance with the development strategy of “bringing in and going out” to promote the total amount of employment in the manufacturing industry and enhance its structure.

(4) Sharing the dividends of technological progress, integrating urban and rural areas, and promoting the common prosperity of all people, The Vision for Socialist Modernization 2035 clearly states that “the gap between urban and rural areas in terms of development and the standard of living of the population will be significantly narrowed”. In promoting the process of realizing common wealth in the new era, the focus should be placed on heterogeneous group differences between urban and rural areas. Narrowing the urban–rural digital divide and accelerating investment in AI equipment. ① In the context of the rural revitalization strategy, AI should be actively promoted in combination with production links in all areas of contemporary agriculture, creating smart equipment, developing smart agriculture and building smart villages, promoting farmers' income growth, and further narrowing the income gap between urban and rural areas; ② Deepening market reforms to promote the rationalization of factor flows. For areas with a low level of development, guide the regulation of labor resource allocation and increase inputs of AI equipment, improve the human resources security system across the board, create a good and fair employment environment, and guarantee equal employment for the labor force so that urban and rural residents can share the opportunities and fruits of development, thereby promoting common prosperity.

## 7 Shortcomings and prospects

There are still two shortcomings in this study. The first is that the definitions of existing studies on AI are diversified and are based on the comprehensive formation of hardware, software, and talents required to achieve AI. The measurement index system needs to be improved and lacks systematic analysis, and the development of AI in China needs to be further analyzed. The second is the impact of AI on the quality of employment. We have only selected income as a reflection of the quality of employment in the manufacturing industry, and have not evaluated the quality of employment from the perspectives of job stability, social security, and social welfare, which is too homogeneous. In the subsequent research, we can further combine the national conditions and economic development strategy of our country to improve the measurement of the development level of AI based on science and rationality. When analyzing the impact of AI on the quality of employment, we can combine objective factors of income and subjective factors, such as job stability, social security, and welfare, to analyze and portray the whole picture. At the same time, it will explore its own development and make a comparative analysis of the economic development model and industrialization process with other developing countries and developed countries to enhance the driving force and resilience of economic development.

## Data availability statement

The original contributions presented in the study are included in the article/supplementary material, further inquiries can be directed to the corresponding author.

## Author contributions

QH: Conceptualization, Data curation, Formal analysis, Methodology, Software, Supervision, Validation, Writing – original draft, Writing – review & editing. JR: Funding acquisition, Formal analysis, Methodology, Project administration, Supervision, Visualization, Writing – review & editing. YC: Conceptualization, Supervision, Validation, Writing – review & editing.

## Footnotes

^1^The formula for the point of inflection is consistent with the parabolic point of inflection calculation, *ê*((−(−0.17/2 × 0.09))) = 2.57.
